# Transspinal direct current stimulation as targeted therapy to increase motor neuron output and restore inhibition in human spinal cord injury

**DOI:** 10.3389/fneur.2026.1773679

**Published:** 2026-03-20

**Authors:** Abdullah M. Sayed Ahmad, Maria Knikou

**Affiliations:** 1 Klab4Recovery Research Program, The City University of New York, New York, NY, United States; 2 Department of Physical Therapy, College of Staten Island, The City University of New York, New York, NY, United States; 3 Biology and Collaborative Neuroscience Program, Graduate Center of the City University of New York, College of Staten Island, New York, NY, United States

**Keywords:** multiple sessions, neuromodulation, neurorecovery, recruitment of motoneurons, spinal cord injury, spinal inhibition, transspinal direct current stimulation

## Abstract

**Introduction:**

In this clinical trial, we evaluated changes in the output of motoneurons and spinal inhibitory mechanisms across multiple spinal segments before and after a series of transspinal direct current stimulation (tsDCS) sessions in humans with and without spinal cord injury (SCI).

**Methods:**

Ten people with chronic SCI and ten healthy participants received daily tsDCS over the low thoracic area while supine with an average stimulation intensity of 2.28 ± 0.02 mA for one hour. Participants with SCI and healthy subjects participated in an average of 15 sessions and 10 sessions, respectively. One day before and 1–2 days after cessation of stimulation sessions, we evaluated the recruitment input–output curves of transspinal evoked potentials (TEPs) and TEPs homosynaptic and postactivation depression in response to low frequency and paired transspinal stimuli.

**Results:**

We found significant changes in the spinal motor output for 10/16 muscles in the American Spinal Injury Association (ASIA) Impairment Scale (AIS) A-B injury, 8/16 muscles in the AIS D, and 10/16 muscles in healthy subjects with facilitation or inhibition to be muscle-dependent. Facilitation was exerted mostly to distal muscles and inhibition to proximal hip muscles. TEPs homosynaptic depression at baseline was similar across subject groups, while TEPs postactivation depression was of lesser strength in the right soleus and medial gastrocnemius in AIS A-B compared to healthy subjects. tsDCS potentiated TEPs postactivation depression of the right soleus and left peroneus longus muscles in AIS A-B but remained unchanged in AIS D and healthy subjects. TEPs homosynaptic depression remained unchanged in all subject groups.

**Discussion:**

This study showed that tsDCS alters the net motor output across multiple spinal segments, potentiates postactivation depression in AIS A-B, and does not affect homosynaptic inhibition in AIS D and healthy subjects. These results provide the first systematic support for tsDCS as a therapeutic intervention in SCI.

## 1 Introduction

Spinal stimulation as a stand-alone or as an adjunct therapeutic modality of activity-based training for restoration of dysfunctional neuronal networks after spinal cord injury (SCI) and other neurological disorders is currently one of the major fields in clinical translational research. Because the spinal cord serves as an integration center for descending and ascending signals, non-invasive transspinal (known also as transcutaneous spinal cord) stimulation has the capacity to induce functional neuroplasticity in distributed neuronal pathways. Several non-invasive protocols with varying intensities, frequencies, montage of electrodes, and polarity spanning from transspinal alternate to transspinal direct current stimulation (tsDCS) have been adopted in animal models and humans (1–4).

tsDCS produces persistent plasticity in distributed motor pathways (5, 6), spinal reflex pathways (7, 8), the motor cortex (9) and sensory processing networks (10), with the electrical field spreading to the sensory ganglia (11). In neurological disorders, including SCI, tsDCS reduces hypertonia and intensity of neuropathic pain, balances deficits, and improves gait speed when administered alone or in combination with other modalities (12, 13). These findings support that tsDCS-induced plasticity is transferred to motor function, thereby bearing great clinical significance as a therapeutic modality. Nonetheless, most evidence derives from studies after a single session of tsDCS. Here we focus on spinal inhibition and recruitment of motoneurons spanning several spinal segments, 1 to 2 days after multiple sessions of cathodal tsDCS in people with and without SCI.

Spinal neuronal organization encompasses a plethora of spinal inhibitory interneurons critical for integration of peripheral sensory and descending inputs during movement (14–17). The spinal interneuronal circuits undergo considerable reorganization after isolated or combined exercise and stimulation training in people with SCI (18–21). One of the spinal inhibitory control mechanisms is homosynaptic depression, which is localized at the Ia-motoneuron presynaptic terminals, manifests as the decreased amplitude of motoneuron depolarization in response to repetitive Ia afferent activation, is maximal at the interstimulus intervals of 1 to 2 s, involves the same afferents mediating the test reflex response, and is stronger in small motoneurons (22). Traditionally, homosynaptic depression is observed on the soleus H-reflex following single-pulse stimulation of the posterior tibial nerve at low frequencies (1.0 Hz compared to 0.2 or 0.1 Hz) or following Achilles tendon vibration (22), a phenomenon reported also for transspinal evoked potentials (TEPs) evoked by transspinal stimulation with alternate current at the thoracolumbar region (23). Homosynaptic depression is greatly impaired and linked to spasticity, spasms, clonus, and poor movement performance in upper motoneuron lesions (24–27).

Alternatively, paired stimuli delivered at a constant stimulation frequency at interstimulus intervals ranging from 50 to 500 ms (termed here postactivation depression) are also used to establish soleus H-reflex or TEP depression in response to repetitive afferent discharges (23, 28). Nonetheless, homosynaptic and postactivation depression of TEPs cannot be ascribed to similar mechanisms as those of the soleus H-reflex because transspinal stimulation produces depolarization of motoneurons over multiple segments and thereby indirectly activates a plethora of interneuronal circuits. Further, manifestation of TEPs coincides with bilateral leg and abdominal muscle contractions, suggesting that recurrent collaterals and Ib inhibitory interneurons are involved. This is supported, via motor unit recordings in humans, by the two distinct phases of inhibitory postsynaptic potentials observed in soleus motoneurons following transspinal stimulation (29).

Motoneurons follow orderly motor-unit recruitment during the graded development of muscle force based on Henneman’s size principle (30, 31). Recruitment of motoneurons manifests in a non-linear sigmoid fashion for descending motor pathways (motor-evoked potential; MEP), spinally mediated reflexes (soleus H-reflex), and peripheral motor axons (M-wave) (32, 33). The non-linear recruitment order reflects the heterogenous nature and distribution of properties of neurons and the heterogeneous distribution of the afferent inputs within a motor pool (34, 35). TEPs recorded bilaterally from ankle and knee muscles are also confined to a non-linear sigmoid recruitment order (36). The main differences between maximal TEPs with H-reflexes and M-waves are that the maximal TEPs likely do not depict recruitment of the whole motoneuron pool to synchronized excitatory Ia afferent volleys but rather represent all excitatory and inhibitory neuronal events for a given motor pool (37, 38); thus, they potentially represent the net spinal motor output from multiple spinal segments.

Collectively, in this study, we assessed changes in the spinal motor output at increasing stimulation intensities and inhibition of motoneurons located at several spinal segments before and after multiple sessions of tsDCS in a cohort of people with and without SCI. The specific objectives of the study were to assess the TEP recruitment curves of 16 muscles, TEPs homosynaptic depression following single-pulse transspinal stimulation at low frequencies (0.1, 0.125, 0.2, 0.33, and 1.0 Hz), and TEPs postactivation depression following transspinal paired stimuli at the interstimulus intervals of 60, 100, 300, and 500 ms before and after multiple sessions of tsDCS. We hypothesized that tsDCS increases the net spinal motor output and regulates spinal inhibitory circuits.

## 2 Materials and methods

### 2.1 Experimental design

In this clinical study/trial, a cohort of eligible people with and without SCI (inclusion and exclusion criteria are presented in Supplementary Table 1) were assigned to receive daily sessions of cathodal tsDCS. During the duration of the study, patients did not receive physical therapy or any other types of intervention.

### 2.2 Participants

All procedures, experiments, and interventions, were approved by the City University of New York Institutional Review Board (IRB) Biomedical Committee (IRB Number 515055) and conducted in accordance with the standards of the Declaration of Helsinki. Ten individuals with chronic SCI (Table 1) and 10 healthy volunteers (seven female; 27.2 ± 5 years, mean ± SD) participated in the clinical study. All participants gave their written informed consent before study enrolment and participation. Five individuals with chronic SCI had American Spinal Injury Association (ASIA) Impairment Scale (AIS) grade D neurological deficit, four had AIS B, and one had AIS A, while the vertebrae level of SCI ranged from Cervical 4 to Thoracic 11. Assessments were scheduled for all subjects in the morning and patients refrained from taking medication that morning.

**Table 1:** Demographic and injury characteristics of participants with chronic spinal cord injury (SCI).

Pub ID	Gender	Age (yrs)	Post injury (yrs)	Level of injury	AIS	Cause of injury	Motor score		# of sessions attended (tsDCS)	Medication
							LL	RL		
R14	M	46	1	T3	A	Skiing	0	0	15	None
R01	M	53	5	C7	B	Ocean wave-related	0	0	17	Baclofen 20 mg 4xD^†^; Cymbalta 60 mg 1xD; Oxybutynin 5 mg 3xD
R06	M	36	4.5	T2	B	MVA	0	0	13	None
R13	M	51	23	T11	B	MVA	2	2	12	Baclofen 20 mg xD
R17	M	31	4	C5	B	Diving into shallows	0	0	14	None
R09	F	20	7	T1	D	SX	24	23	16	None
R11	M	39	7	T9	D	GSW	25	25	15	Gabapentin 800 mg 3xD; Baclofen 10 mg 3xD^‡^
R16	M	57	4	C4	D	Fall & spinal stenosis	23	21	15	Gabapentin 400 mg 4xD; Baclofen 10 mg 5xD; Oxybutynin 10 3xD; Oxycontin 5 mg 2xD
R18	M	61	17	C5-6	D	MVA	25	25	15	Synthroid 225 mg 1Xd
R19	M	60	2	C6	D	Fall	18	17	15	Oxybutynin 10 mg 2x/day; Pravachol 40 mg 1x/day

The level of injury corresponds to the vertebral level. Following the American Spinal Injury Association (ASIA) impairment scale (AIS) assessment, motor scores are indicated based on the manual muscle test of five key muscles and evaluated as 5 = normal muscle power, 4 = active movement against gravity with slight resistance, 3 = active movement against gravity, 2 = active movement with gravity eliminated, 1 = trace muscle contraction, 0 = no contraction. M: male; F: female; C: cervical; T: thoracic; MVA = Motor vehicle accident; SX = Surgery; GSW = Gunshot wound; xD = Times daily. ^†^Participant delayed ingestion of baclofen until after each experiment. ^‡^Participant ceased baclofen prior to the duration of the study. ^¥^Participant ceased baclofen after the first day of experiments.

### 2.3 Surface electromyography

Surface electromyographic (EMG) activity was recorded bilaterally by surface single-dipole electrodes (MA400-28 16 EMG channels, Motion Lab Systems, Lake Elsinore, CA, United States) from the vastus lateralis (VL), medial hamstrings (MH), lateral hamstrings (LH), gracilis (GRC), soleus (SOL), medial gastrocnemius (MG), peroneus longus (PL), and tibialis anterior (TA) muscles. EMG signals were amplified, filtered (10–1,000 Hz), sampled at 2000 Hz with 1,401 POWER (Cambridge Electronics Design Ltd., Cambridge, United Kingdom), and stored for offline analysis.

### 2.4 Intervention: multiple sessions of tsDCS

We administered cathodal tsDCS because of the known greater effects compared to anodal tsDCS (5, 39, 40). tsDCS was delivered using a direct current stimulator (neuroConn DC stimulator PLUS, neuroCare Group GmbH, München, Germany). A rubber electrode (cathode; 3.2 cm x 3.2 cm; Amrex-Zetron Inc., California, United States) was housed in an active square steel mesh and covered by a sponge soaked in 0.9% saline solution. The electrode was placed centrally over Thoracic 10 to 12 vertebrae. The position was determined via manual palpation of the spinal processes starting from Cervical 7, confirmed two vertebrae above the attachment point of the twelfth rib, and marked with a non-toxic surgical pen for constancy across stimulation sessions. The anode (same type as the cathode; 3.2 cm x 3.2 cm) was placed on the abdomen left of the umbilicus to avoid vital organs. This montage produces maximum electric field potentials in a longitudinal direction along the spinal cord (41).

For all subjects, tsDCS was delivered daily during weekdays, excluding holidays, while the subject was lying supine with knee and hip joints flexed at 30° and supported by pillows and bolsters at the legs to minimize hip external rotations. Subjects with SCI received an average of 14.67 ± 0.47 stimulation sessions for 50.25 ± 2.25 min per session (Table 1). Healthy subjects received 10 stimulation sessions for an average of 44.96 ± 0.27 min per session. Depending on the subjects’ availability, additional or fewer sessions were performed to ensure that the neurophysiological recordings post intervention did not occur after a weekend.

Every 5 to 10 min, the stimulation intensity was ramped down slowly to 0 mA and maintained for 20 s followed by a slow ramp up to the designated intensity established largely based on self-reported discomfort levels. This was repeated as many times as needed until a total of 45 min of stimulation was administered. This approach was also chosen to limit skin irritations. The stimulation intensity ranged between 1.25 and 3.0 mA. The average stimulation intensity administered was 2.28 ± 0.02 mA. Both subject groups received similar intensities across the entire intervention despite the small increases and decreases. The intensity used was within the safety limits of 2.3 mA/cm^3^ for current density threshold during invasive spinal stimulation and 25 mA/cm^3^ for pulse electrical stimulation known to cause tissue damage (42–44). Due to the intensity of stimulation delivered, it was impossible to blind the participants to the sessions and therefore no sham group was included in the study. No significant changes were noted in the blood pressure of any participant during the stimulation sessions and/or experiments. The major complaint was skin redness or irritation that subsided within a few hours, while some developed a mild skin rash due to the daily application of the saline solution. Tingling and burning or itching sensations during the ramp-up and down phases of stimulation were also reported. Lastly, mild back and neck pain possibly due to position requirements of the study and a mild–moderate but transient headache was also reported.

### 2.5 Assessment of neurophysiological biomarkers before and 1–2 days after multiple sessions of tsDCS

Neurophysiological biomarkers in all subjects were tested 1 day before and 1–2 days after tsDCS stimulation sessions. Transspinal stimulation over the thoracolumbar region with alternate current via monophasic pulses of 1 ms duration in relaxed supine subjects was performed according to procedures we have previously used extensively in our laboratory (33, 36, 45, 46). The Thoracic 10 spinous process was identified via palpation and in consolidation with anatomical landmarks. A single cathode electrode (Uni-Patch™ EP84169, 10.2 × 5.1 cm^2^, MA, United States) was placed along the vertebrae equally between the left and right paravertebral sides. The electrode covered Thoracic 10 to Lumbar 1–2 vertebral levels. These vertebral levels correspond to Lumbar 1 and Sacral 2 spinal segments and thus to the segmental innervation of the muscles from which compound muscle action potentials (e.g., TEPs) were recorded. A pair of interconnected reusable self-adhered anode electrodes (same type as the cathode; 10.2 × 5.1 cm^2^) were placed on either side of the umbilicus or bilaterally on the iliac crests depending on the participant’s level of comfort or if the stimulation caused bladder discomfort (35). The cathode and anode electrodes were connected to a constant current stimulator (DS7A, Digitimer, United Kingdom) that was triggered by Spike 2 scripts (Cambridge Electronics Design Ltd., United Kingdom). Optimal electrode placement was based on the presence of TEPs bilaterally in distal lower limb muscles at low stimulation intensities. Once the optimal location was identified, the electrodes were affixed to the skin via Tegaderm transparent film (3 M Healthcare, St Paul, Minnesota, United States).

Transspinal stimulation was delivered randomly within and across subjects at (1) increasing intensities to assemble the TEP recruitment curves of 16 muscles at 0.2 Hz; (2) 0.1, 0.125, 0.2, 0.33, and 1.0 Hz to establish changes in TEP low-frequency homosynaptic depression; and (3) following paired transspinal stimuli at the interstimulus intervals of 60, 100, 300, and 500 ms at 0.2 Hz to establish changes in TEPs postactivation depression. TEPs were recorded at 1.2 times the right SOL TEP threshold and corresponded to 20.6 ± 6.5 mA for AIS A-B, 23.3 ± 5.3 mA for AIS D, and 17.8 ± 1.84 mA for healthy subjects. For homosynaptic depression, 15 TEPs were recorded at each stimulation frequency. For postactivation depression, 15 pairs of TEPs were recorded at each interstimulus interval. Lastly, at least 100 TEPs were recorded for the recruitment curves. To ensure the same position of the cathode electrode before and after tsDCS, the position was marked with a hypoallergic pen.

### 2.6 Data analysis and statistics

All data in Spike 2 software were saved with a code to minimize bias and protect personal health information. Only after the data were analyzed were they linked to healthy subject groups or to a specific type of AIS injury. TEPs recorded from each muscle were measured as the area under the full-wave-rectified waveform within identical time windows (Spike 2, Cambridge Electronics Design Ltd., U. K.). TEPs recorded following single-pulse transspinal stimulation at increasing stimulation intensities to assemble the recruitment input–output curves were normalized to the associated maximal TEP (TEPmax) observed at the recording session before tsDCS sessions. For each subject, muscle, and time of testing, the normalized TEP amplitudes were plotted against the non-normalized stimulation intensities, and a Boltzmann sigmoid function (Equation 1; SigmaPlot 11, Systat Software Inc.) was fitted to the data. In Equation 1, *m* is the slope parameter of the function, *S50-TEPmax* is the stimulus required to elicit a TEP equivalent to 50% of the TEPmax, and *s* is the TEP amplitude at a given stimulus value TEP.


$$\mathrm{TEP}\left(s\right)=\frac{\mathrm{TEPmax}}{\left(1+\exp\left(m\left(s50-s\right)\right)\right)\;}$$ (1)

For each TEP, the predicted S50-TEPmax observed before and after multiple sessions of tsDCS was used to normalize the associated stimulation intensities. Averages of normalized TEPs were calculated in steps of 0.05 from 0.2 up to 1.6 or 1.7 times the S50-TEPmax. Repeated measures ANOVA were then performed to the normalized TEPs grouped at multiples of S50-TEPmax before and after tsDCS to establish the main effects of time. When a main effect was found, Holm-Sidak tests for multiple comparisons were used to test significant interactions between time and intensities as multiples of S50-TEPmax.

For each subject, muscle, and time of testing TEPs evoked at 0.125, 0.2, 0.33, and 1.0 Hz were expressed as a percentage of the mean amplitude of the homonymous TEP evoked at 0.1 Hz. Similarly, TEPs evoked by the second transspinal stimulus in the paired paradigm at the interstimulus intervals of 60, 100, 300, and 500 ms were normalized to the mean amplitude of the homonymous TEP observed with the first transspinal stimulus. For both neurophysiological measures, mixed linear model analysis, including all normalized TEPs recorded from each subject, with factors time, interstimulus intervals or stimulation frequencies and subject ID was performed to establish the main and interaction effects. Bonferroni pairwise multiple comparisons were performed only for muscles that showed statistically significant effects. Further, to determine differences or similarities on the strength of homosynaptic depression among subjects’ groups, the TEPs recorded at baseline were grouped based on stimulation frequency and subject group. A linear mixed model analysis was then applied to the data. The same statistical analysis was performed for TEPs postactivation depression with the data grouped based on interstimulus interval and subject group. Values were considered significant when *p* < 0.05. All results are presented as mean ± SD.

## 3 Results

### 3.1 Motoneuron output spanning multiple spinal segments

**Figure 1:**
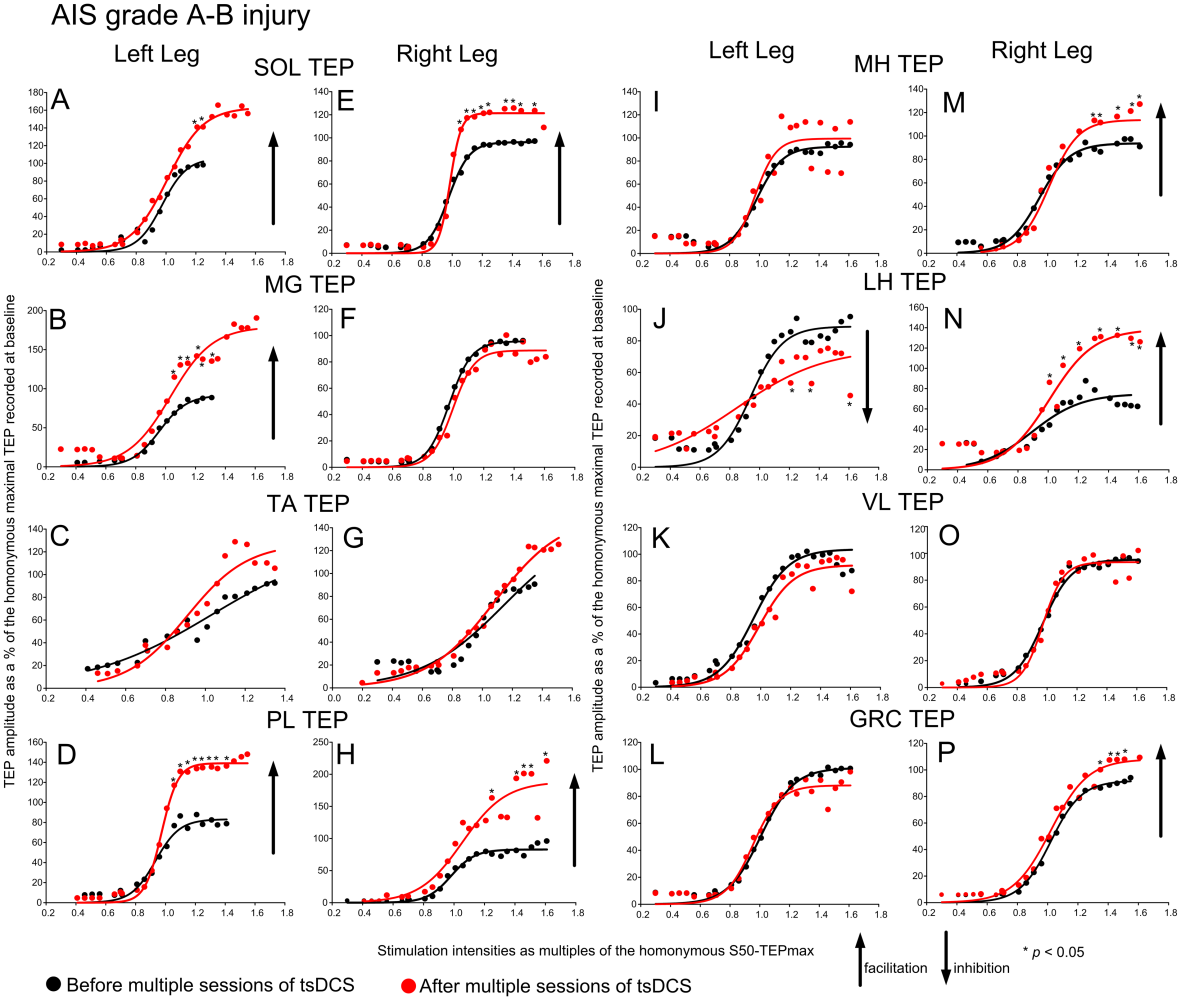
Recruitment of motoneurons in AIS grade A-B injury before and after tsDCS. Transspinal evoked potentials (TEPs) recruitment input-output curves from the left and right SOL **(A, E)**, MG **(B, F)**, TA **(C, G)**, PL **(D, H)**, MH **(I, M)**, LH **(J, N)**, VL **(K, O)**, and GRC **(L, P)** muscles from all subjects with AIS grade A-B injuries before (black circles) and 1-2 days after (red circles) an average of 15 sessions of tsDCS. The corresponding sigmoid function fitted to the responses are shown. The TEPs were normalized to the homonymous maximal TEP recorded at baseline and grouped in multiples of stimulation intensities that were normalized to the intensity corresponding to the homonymous 50 % of the maximal TEP. Arrows indicate inhibition or facilitation based on 2-way repeated measures ANOVA with levels time and normalized intensities, while asterisks denote the data point that statistically significant differences before and after tsDCS were found based on Bonferroni pairwise comparison results. SOL: soleus; MG: medial gastrocnemius; TA: tibialis anterior; PL: peroneus longus; MH: medial hamstrings; LH: lateral hamstrings; VL: vastus lateralis; GRC: gracilis.

To assess changes in the net spinal motor output at multiple spinal segments, the TEP recruitment input–output curves were assembled in all subjects while supine before and 1–2 days after multiple sessions of tsDCS. The TEPs recorded from all muscles in subjects with *AIS grade A-B injury*, as percentages of the homonymous TEP recorded at baseline and plotted against multiples of the homonymous S50-TEPmax, along with the sigmoid fits, are depicted in Figure 1. A significant effect before and after tsDCS was found for the left SOL (F_1,125_ = 8.55, *p* = 0.004; 2-way rmANOVA; TEPs were grouped across subjects from 0.46 to 1.31 x S50-TEPmax), right SOL (F_1,118_ = 52.24, *p* < 0.001), left MG (F_1,113_ = 14.36, *p* < 0.001), left PL (F_1,116_ = 15.63, *p* < 0.001), right PL (F_1,143_ = 10.9, *p* = 0.001), right MH (F_1,126_ = 3.51, *p* < 0.001), left LH (F_1,126_ = 4.61, *p* < 0.001), right LH (F_1,115_ = 10.7, *p* < 0.001), and right GRC (F_1,123_ = 11.93, *p* < 0.001) TEP recruitment curves. Asterisks in Figure 1 indicate the data points that statistically significant differences before and after tsDCS were found on based on post-hoc Bonferroni tests. In contrast, no significant changes were found for the right MG (F_1,116_ = 0.22, *p* = 0.421), left TA (F_1,143_ = 0.32, *p* = 0.251), right TA (F_1,143_ = 0.12, *p* = 0.826), left MH (F_1,112_ = 0.003, *p* = 0.95), right VL (F_1,142_ = 0.57, *p* = 0.44), left VL (F_1,115_ = 0.4, *p* = 0.47), or left GRC (F_1,128_ = 0.32, *p* = 0.57) TEP recruitment curves.

**Figure 2:**
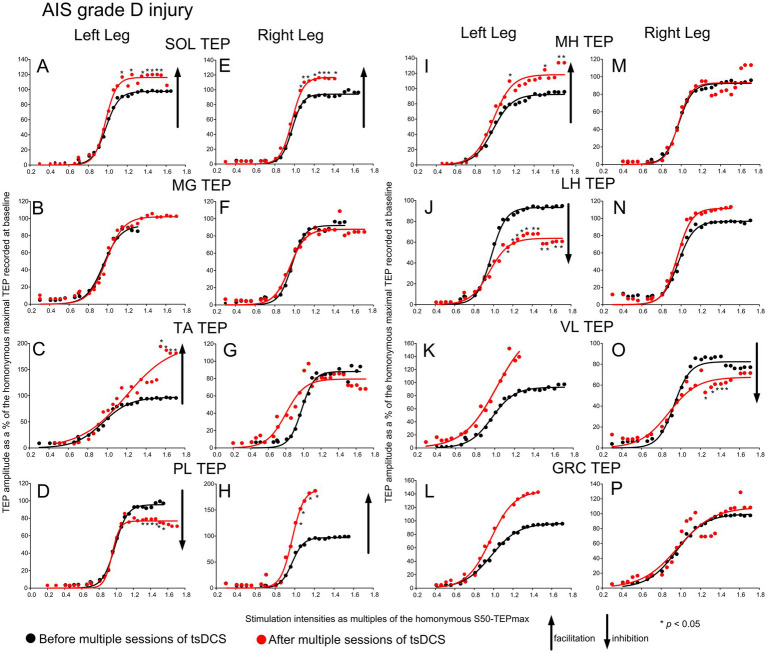
Recruitment of motoneurons in AIS grade D injuries before and after tsDCS. Transspinal evoked potentials (TEPs) recruitment input-output curves from the left and right SOL **(A, E)**, MG **(B, F)**, TA **(C, G)**, PL **(D, H)**, MH **(I, M)**, LH **(J, N)**, VL **(K, O)**, and GRC **(L, P)** muscles from all subjects with AIS grade D injuries before (black circles) and 1-2 days after (red circles) an average of 15 sessions of tsDCS. The corresponding sigmoid function fitted to the responses are shown. The TEPs were normalized to the homonymous maximal TEP recorded at baseline and grouped in multiples of stimulation intensities normalized to the homonymous 50 % of the maximal TEP. Arrows indicate inhibition or facilitation based on 2-way repeated measures ANOVA with levels time and normalized intensities, while asterisks denote the data point that statistically significant differences before and after tsDCS were found based on Bonferroni pairwise comparison results. SOL: soleus; MG: medial gastrocnemius; TA: tibialis anterior; PL: peroneus longus; MH: medial hamstrings; LH: lateral hamstrings; VL: vastus lateralis; GRC: gracilis.

The TEPs recorded from all muscles in subjects with *AIS grade D injury* along with the sigmoid fits are depicted in Figure 2. tsDCS produced significant changes in left SOL (F_1,143_ = 41.3, *p* < 0.001; 2-way rmANOVA for TEPs grouped across subjects from 0.71 to 1.71 x S50-TEPmax), right SOL (F_1,131_ = 21.65, *p* < 0.001), left TA (F_1,118_ = 27.63, *p* < 0.001), left PL (F_1,135_ = 9.9, *p* = 0.002), right PL (F_1,142_ = 15.99, *p* < 0.001), left MH (F_1,152_ = 61.85, *p* < 0.001), left LH (F_1,151_ = 146.79, *p* < 0.001) and right VL (F_1,137_ = 18.61, *p* < 0.001) TEP recruitment curves. In contrast, no significant changes were found for the left MG (F_1,129_ = 0.48, *p* = 0.48), right MG (F_1,127_ = 1.2, *p* = 0.29), right TA (F_1,148_ = 0.57, *p* = 0.44), right MH (F_1,145_ = 0.009, *p* = 0.92), right LH (F_1,145_ = 2.44, *p* = 0.12), left VL (F_1,134_ = 0.1, *p* = 0.74), left GRC (F_1,144_ = 3.59, *p* = 0.06), or right GRC (F_1,125_ = 0.55, *p* = 0.45) TEP recruitment curves. Asterisks in Figure 2 indicate the data points that statistically significant differences before and after tsDCS were found on based on post-hoc Bonferroni tests.

**Figure 3:**
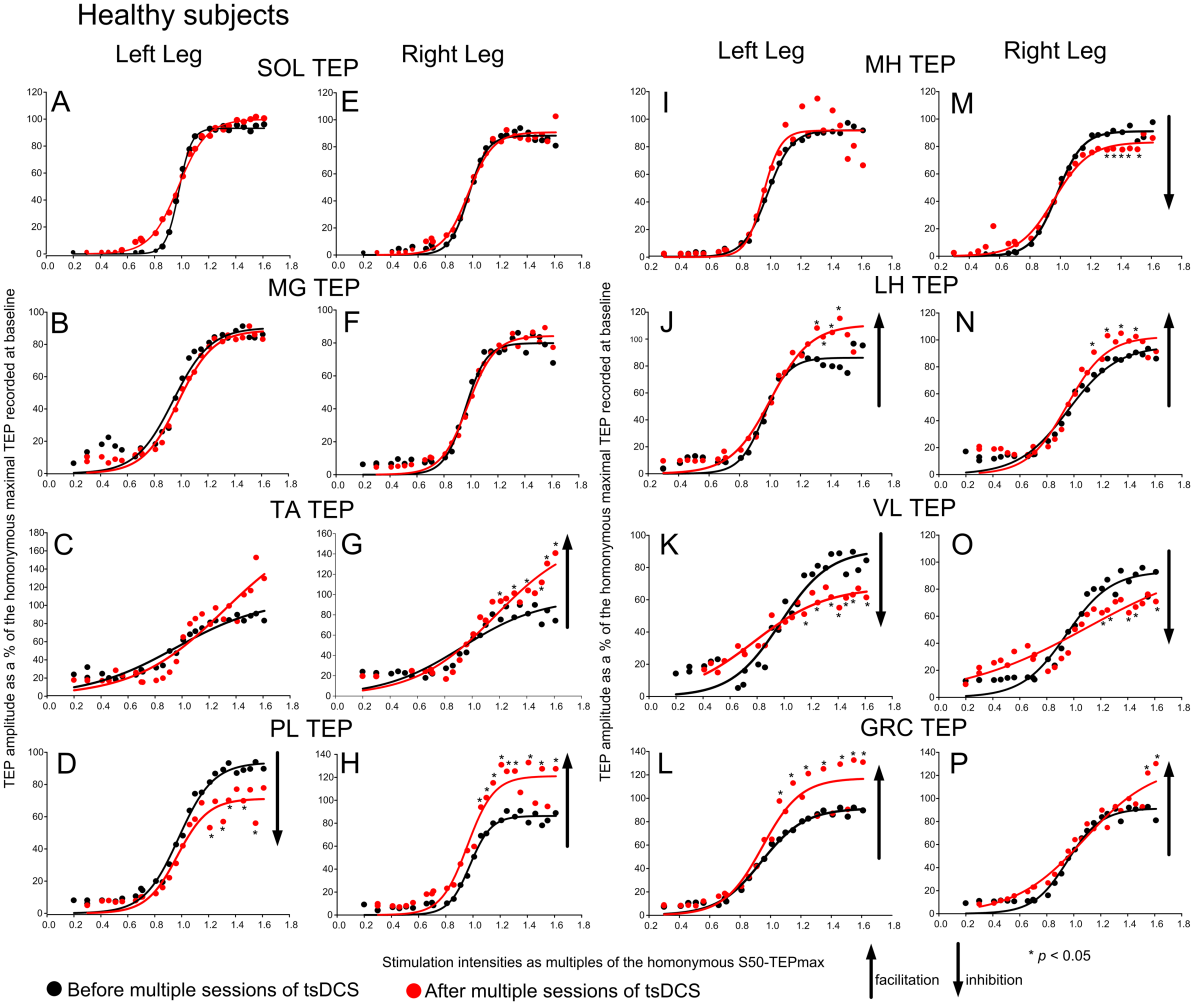
Recruitment of motoneurons in healthy subjects before and after tsDCS. Transspinal evoked potentials (TEPs) recruitment input-output curves from the left and right SOL **(A, E)**, MG **(B, F)**, TA **(C, G)**, PL **(D, H)**, MH **(I, M)**, LH **(J, N)**, VL **(K, O)**, and GRC **(L, P)** muscles from all healthy subjects before (black circles) and 1-2 days after (red circles) 10 sessions of tsDCS. The corresponding sigmoid function fitted to the responses are shown. The TEPs were normalized to the homonymous maximal TEP recorded at baseline and grouped in multiples of stimulation intensities normalized to the homonymous 50 % of the maximal TEP. Arrows indicate inhibition or facilitation based on 2-way repeated measures ANOVA with levels time and normalized intensities, while asterisks denote the data point that statistically significant differences before and after tsDCS were found based on Bonferroni pairwise comparison results. SOL: soleus; MG: medial gastrocnemius; TA: tibialis anterior; PL: peroneus longus; MH: medial hamstrings; LH: lateral hamstrings; VL: vastus lateralis; GRC: gracilis.

The TEPs recorded from all muscles in *healthy subjects* along with the sigmoid fits are shown in Figure 3. A statistically significant effect before and after tsDCS was found for the right TA (F_1,240_ = 12.61, *p* < 0.001; 2-way rmANOVA for TEPs grouped across subjects from 0.66 to 1.61 x S50-TEPmax), left PL (F_1,255_ = 35.58, *p* < 0.001), and right PL (F_1,242_ = 22.49, *p* < 0.001) TEP recruitment curves. In contrast, no significant changes were found as a function of time for the left SOL (F_1,196_ = 3.31, *p* = 0.07), right SOL (F_1,233_ = 1.25, *p* = 0.26), left MG (F_1,220_ = 2.31, *p* = 0.31), right MG (F_1,226_ = 0.22, *p* = 0.63), or left TA (F_1,241_ = 3.73, *p* = 0.055) TEP recruitment curves. Regarding the TEP recruitment curves recorded from knee muscles, a significant effect before and after tsDCS was found for the right MH (F_1,234_ = 8.52, *p* < 0.004), left LH (F_1,244_ = 5.7, *p* = 0.018), right LH (F_1,243_ = 9.94, *p* = 0.002), left VL (F_1,130_ = 14.77, *p* < 0.001), right VL (F_1,111_ = 11.11, *p* < 0.001), left GRC (F_1,209_ = 23.25, *p* < 0.001), and right GRC (F_1,187_ = 5.61, *p* = 0.019). No significant effects before or after tsDCS were found for the left MH (F_1,247_ = 0.83, *p* = 0.36) TEP recruitment curves. Asterisks in Figure 3 indicate the data points that statistically significant differences before and after tsDCS were found on based on post-hoc Bonferroni tests.

### 3.2 Reorganization of postactivation depression

**Figure 4:**
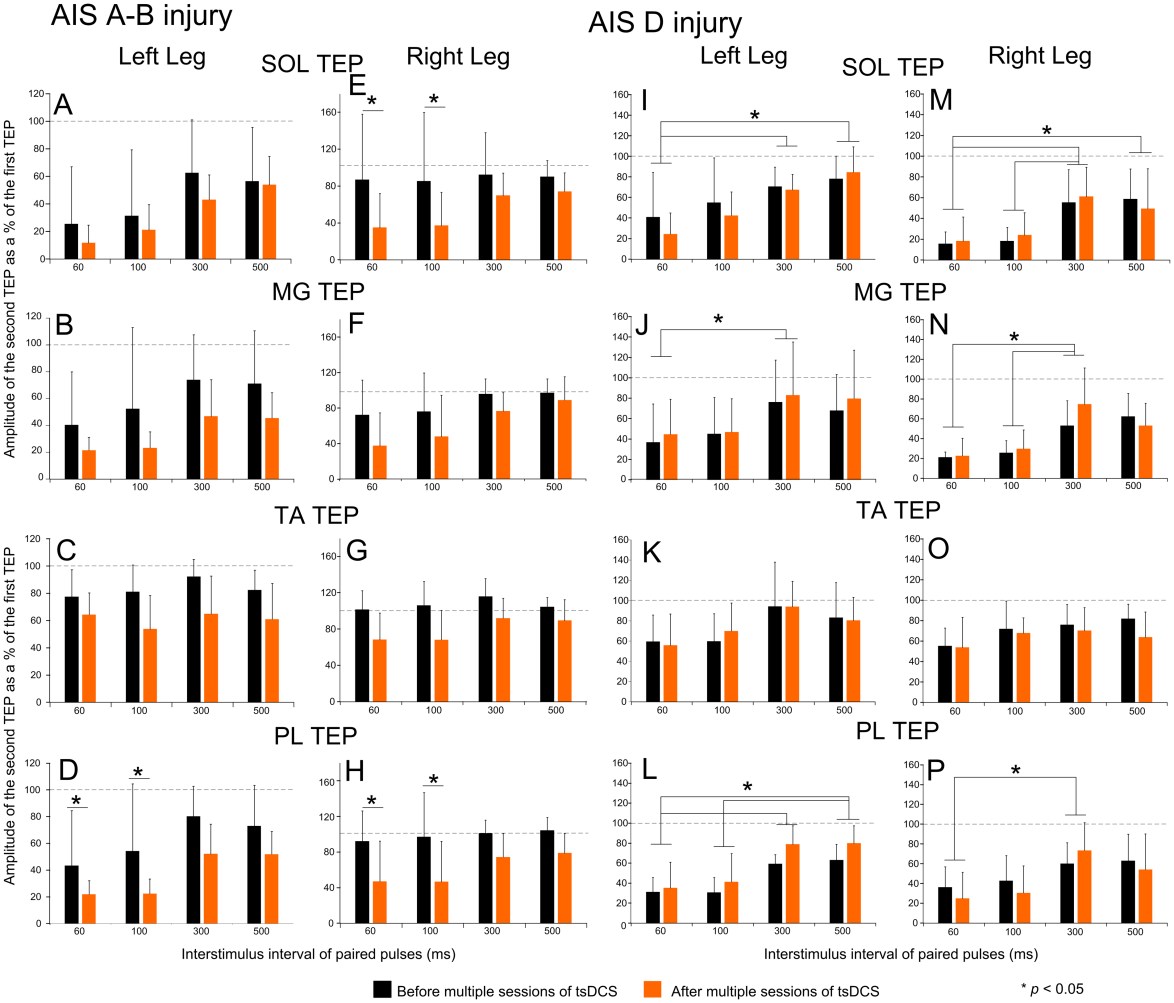
Postactivation depression before and after tsDCS in people with SCI. Transspinal evoked potentials (TEPs) evoked at different interstimulus intervals of paired pulses before (black) and after (orange) multiple sessions of cathodal tsDCS are indicated for the left and right SOL **(A, E)**, MG **(B, F)**, TA **(C, G)**, and PL **(D, H)** muscles for AIS A-B subjects, and for the left and right SOL **(I, M)**, MG **(J, N)**, TA **(K, O)**, and PL **(L, P)** muscles for and AIS D subjects. On the abscissa, the interstimulus interval of paired pulses in ms is indicated. Ordinate indicates the TEP amplitude evoked by the second paired pulse normalized to the homonymous TEP evoked by the first pulse. Asterisks indicate statistically significant differences among different interstimulus intervals or before vs. after tsDCS based on Bonferroni pairwise comparison results. Error bars denote the SD. SOL: soleus; MG: medial gastrocnemius; TA: tibialis anterior; PL: peroneus longus.

The TEP evoked by the second paired pulse delivered at 60, 100, 300, and 500 ms interstimulus intervals as a percentage of the homonymous TEP evoked with the first pulse, which represents TEPs postactivation depression, for all ankle muscles in all subjects with *AIS A-B injury* is indicated in Figures 4A–H. Linear factorial mixed model analysis showed no significant differences among interstimulus intervals for all (*p* > 0.05) except the left SOL (*p* = 0.017), left PL (*p* = 0.01), and right PL (*p* = 0.039) TEPs, supporting the moderate (in three out of eight muscles) presence of postactivation depression in this subject group. Significant differences before and after intervention were found for the left PL (F_2.74_ = 15.24, *p* = 0.035), right SOL (F_2.74_ = 11.68, *p* = 0.048), and right PL (F_2.74_ = 19.07, *p* = 0.027) TEPs.

The TEPs postactivation depression for all subjects with *AIS D injury* and ankle muscles before and after multiple sessions of tsDCS is indicated in Figures 4I–P. Linear mixed model analysis for all TEPs recorded from ankle extensors showed significantly different response amplitudes among interstimulus intervals (*p* < 0.05), while no significant differences among interstimulus intervals was found for the left and right TA TEPs (*p* > 0.05). Absent postactivation depression bilaterally in TA muscles may be related to the types of TA motoneurons recruited by transspinal stimulation. These findings support a strong presence of TEP postactivation depression (in six out of eight muscles) in this subject group. For the left SOL TEP, no significant differences before or after tsDCS (F_2.67_ = 0.77, *p* = 0.45) or significant interactions between time and interstimulus intervals (F_6.02_ = 2.41, *p* = 0.16) were found. Similar results were found for before and after tsDCS for the left MG (F_2.67_ = 0.006, *p* = 0.94), left TA (F_2.67_ = 0.004, *p* = 0.95), left PL (F_2.67_ = 2.49, *p* = 0.22), right SOL (F_2.67_ = 0.01, *p* = 0.9), right MG (F_2.67_ = 0.27, *p* = 0.64), right TA (F_2.67_ = 0.48, *p* = 0.54), and right PL (F_2.67_ = 0.33, *p* = 0.6) TEPs, providing evidence that tsDCS did not affect the amount of postactivation depression in participants with AIS D injury.

**Figure 5:**
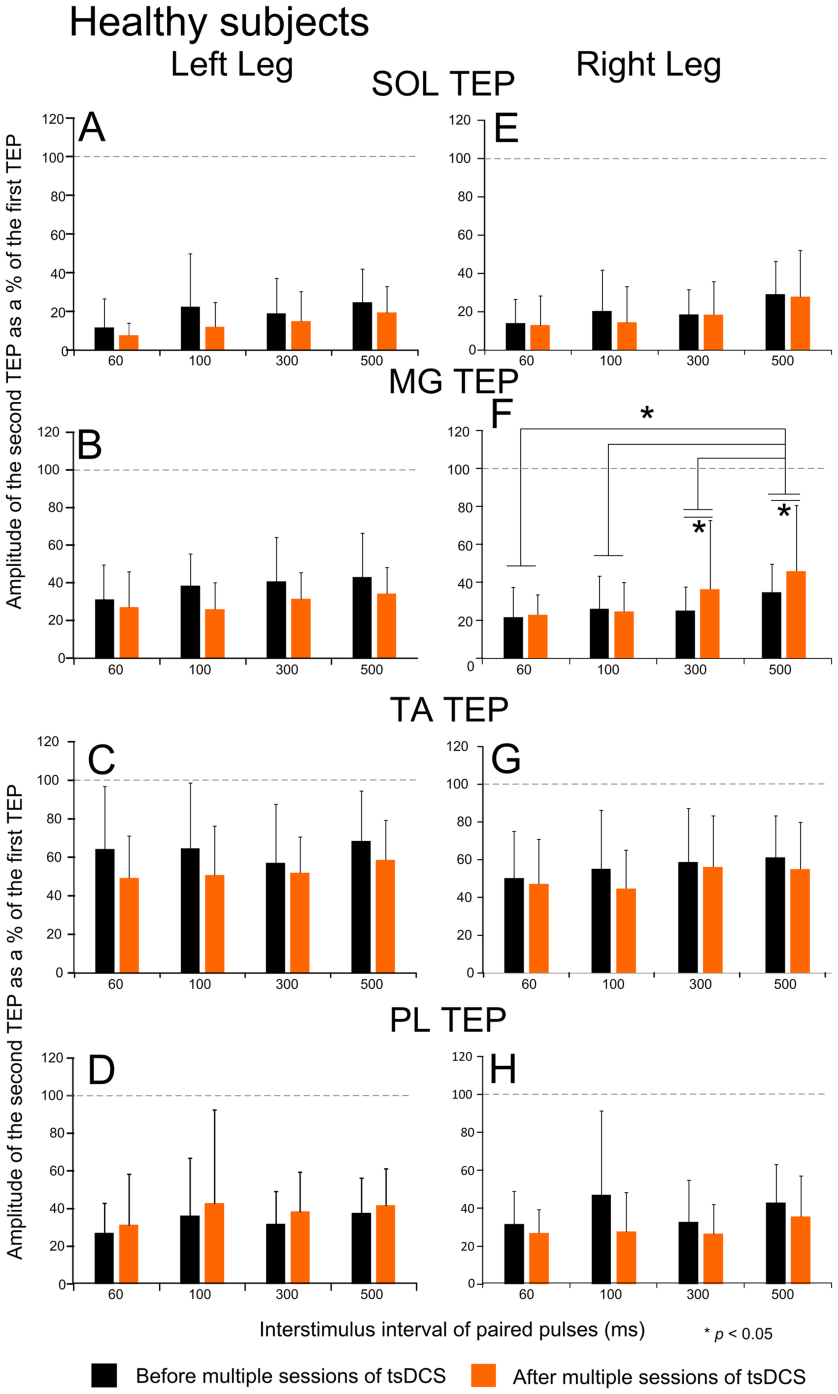
Postactivation depression before and after tsDCS in healthy subjects. Transspinal evoked potentials (TEPs) evoked at different interstimulus intervals of paired pulses before (black) and after (orange) multiple sessions of cathodal tsDCS are indicated for the left and right SOL **(A, E)**, MG **(B, F)**, TA **(C, G)**, and PL **(D, H)** muscles for all healthy subjects. On the abscissa, the interstimulus interval of paired pulses in ms is indicated. Ordinate indicates the TEP amplitude evoked by the second paired pulse normalized to the homonymous TEP evoked by the first pulse. Asterisks indicate statistically significant differences for the MG TEP evoked at different interstimulus intervals and before vs. after intervention at 300 and 500 ms based on Bonferroni pairwise comparison results. No other significant differences were found. Error bars denote the SD. SOL: soleus; MG: medial gastrocnemius; TA: tibialis anterior; PL: peroneus longus.

The TEPs postactivation depression for all *healthy subjects* and ankle muscles before and after multiple sessions of tsDCS is indicated in Figure 5. Linear mixed model analysis did not show statistically significant different TEP amplitudes among interstimulus intervals (*p* > 0.05) for all TEPs except for the left MG (F_860_ = 47.53, *p* < 0.001). This finding supports that the TEPs postactivation depression in all ankle muscles is of similar strength among all interstimulus intervals tested. Further, postactivation depression in the left SOL TEP did not change after intervention (F_2.78_ = 0.98, *p* = 0.37), while a significant interaction between time and interstimulus intervals was not found (F_6.08_ = 0.16, *p* = 0.91). Similar results were found for before and after intervention for the left TA (F_2.79_ = 1.5, *p* = 0.31), left PL (F_2.79_ = 0.57, *p* = 0.5), right SOL (F_2.79_ = 0.57, *p* = 0.5), right MG (F_2.79_ = 0.4, *p* = 0.57), right TA (F_2.79_ = 0.46, *p* = 0.54), and right PL (F_2.79_ = 1.72, *p* = 0.28) TEPs. In contrast, a significant effect of time (F_860_ = 36.12, *p* < 0.001) was found only for the left MG TEP, which displayed decreased depression after tsDCS. These results strongly support the inability of multiple sessions of tsDCS to alter the strength of TEPs postactivation depression in healthy subjects. Lastly, linear mixed model analysis showed statistically significant differences among subject groups for the right SOL (F_4.72_ = 6.14, *p* = 0.04; AIS A-B vs. healthy controls) and right MG (F_4.72_ = 9.09, *p* = 0.02; AIS A-B vs. healthy controls) TEPs postactivation depression recorded at baseline at different interstimulus intervals. However, this was not the case for the left SOL (F_4.72_ = 1.64, *p* = 0.28), left MG (F_4.72_ = 0.92, *p* = 0.457), left TA (F_4.72_ = 0.53, *p* = 0.61), left PL (F_4.72_ = 1.63, *p* = 0.28), right TA (F_4.72_ = 4.19, *p* = 0.09), or right PL (F_4.72_ = 5.03, *p* = 0.067) TEPs postactivation depression.

### 3.3 Reorganization of homosynaptic depression

**Figure 6:**
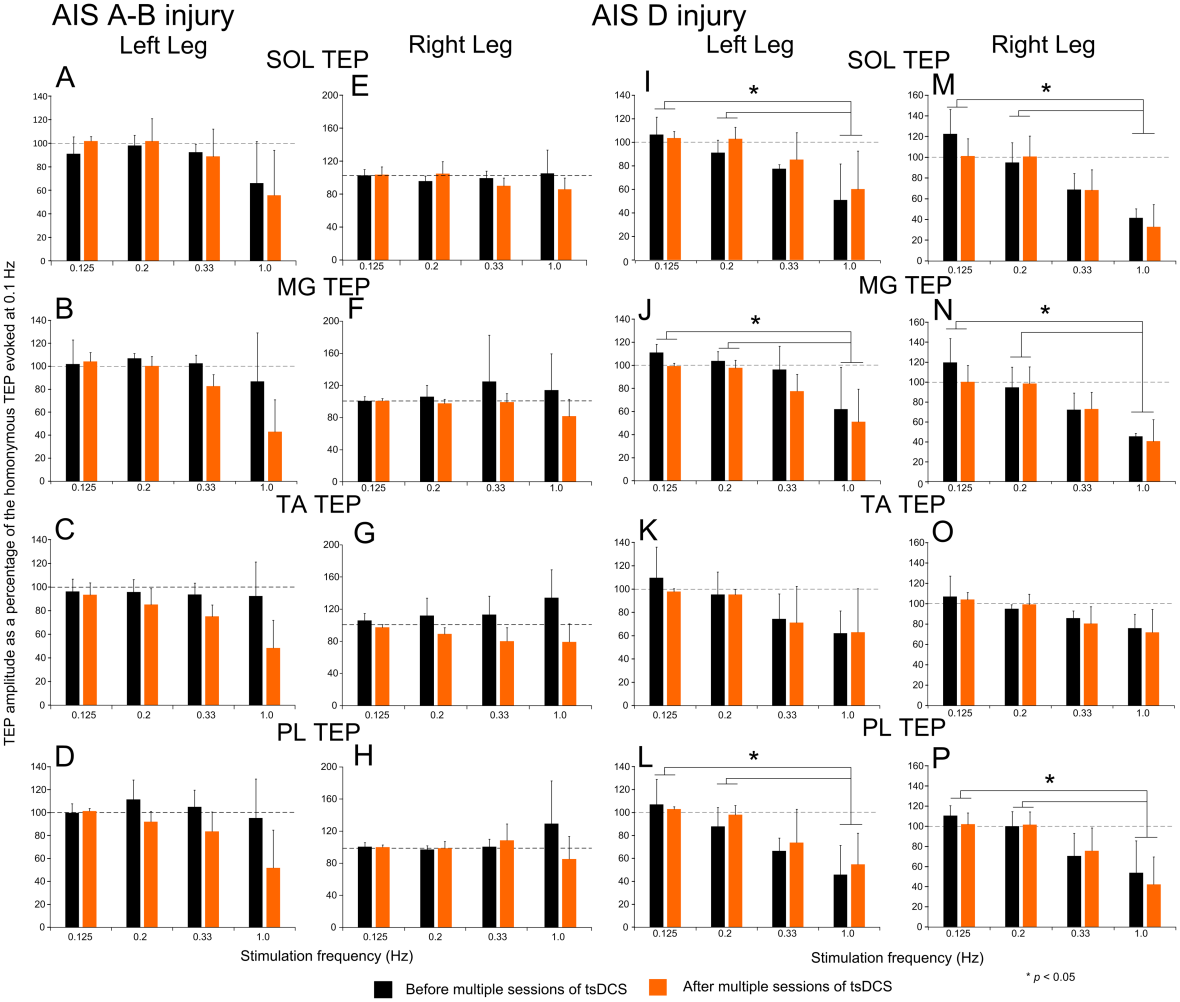
Homosynaptic depression before and after tsDCS in people with SCI. Transspinal evoked potentials (TEPs) evoked at different stimulation frequencies before (black) and after (orange) multiple sessions of cathodal tsDCS are indicated for the left and right SOL **(A, E)**, MG **(B, F)**, TA **(C, G)**, and PL **(D, H)** muscles for AIS A-B subjects, and for the left and right SOL **(I, M)**, MG **(J, N)**, TA **(K, O)**, and PL **(L, P)** muscles for AIS D subjects. On the abscissa, the stimulation frequency is indicated. Ordinate indicates the TEPs amplitude normalized to the homonymous TEP evoked at a stimulation frequency of 0.1 Hz. Asterisks indicate statistically significant differences for TEPs evoked at different stimulation frequencies based on Bonferroni pairwise comparison results. No significant differences were found before and after tsDCS. Error bars denote the SD. SOL: soleus; MG: medial gastrocnemius; TA: tibialis anterior; PL: peroneus longus.

The TEP amplitudes evoked at different stimulation frequencies (0.125, 0.2, 0.33, and 1.0 Hz) as a percentage of the homonymous TEP evoked at 0.1 Hz, which represents the strength of homosynaptic depression, for all subjects with *AIS A-B* injury and ankle muscles is indicated in Figures 6A–H. Linear factorial mixed model analysis showed no significant differences among stimulation frequencies tested (*p* > 0.05), with TEPs at 1.0 Hz reaching amplitudes similar to those observed at 0.1 Hz. Further, no significant differences before and after tsDCS were found (*p* > 0.05) for all TEPs.

The TEP homosynaptic depression from all subjects with *AIS D* injury is indicated in Figures 6I–P. Linear factorial mixed model analysis showed significant differences between 1.0 Hz vs. 0.125 Hz and 1.0 Hz vs. 0.2 Hz for the left and right SOL, MG, and PL TEPs (*p* < 0.05; Bonferroni pairwise comparisons) but not for the left (F_6.17_ = 1.92, *p* = 0.22) and right (F_6.18_ = 2.24, *p* = 0.18) TA TEPs. Additionally, no statistically significant differences before and after tsDCS were found for the left SOL TEP (F_2.8_ = 1.77, *p* = 0.28), a result observed for all remaining TEPs (*p* > 0.05). These results suggest absent TEPs homosynaptic depression in *AIS A-B* but a strong presence in *AIS D* injuries, as well as the inability of tsDCS to strengthen or alter homosynaptic depression in either SCI subject groups.

**Figure 7:**
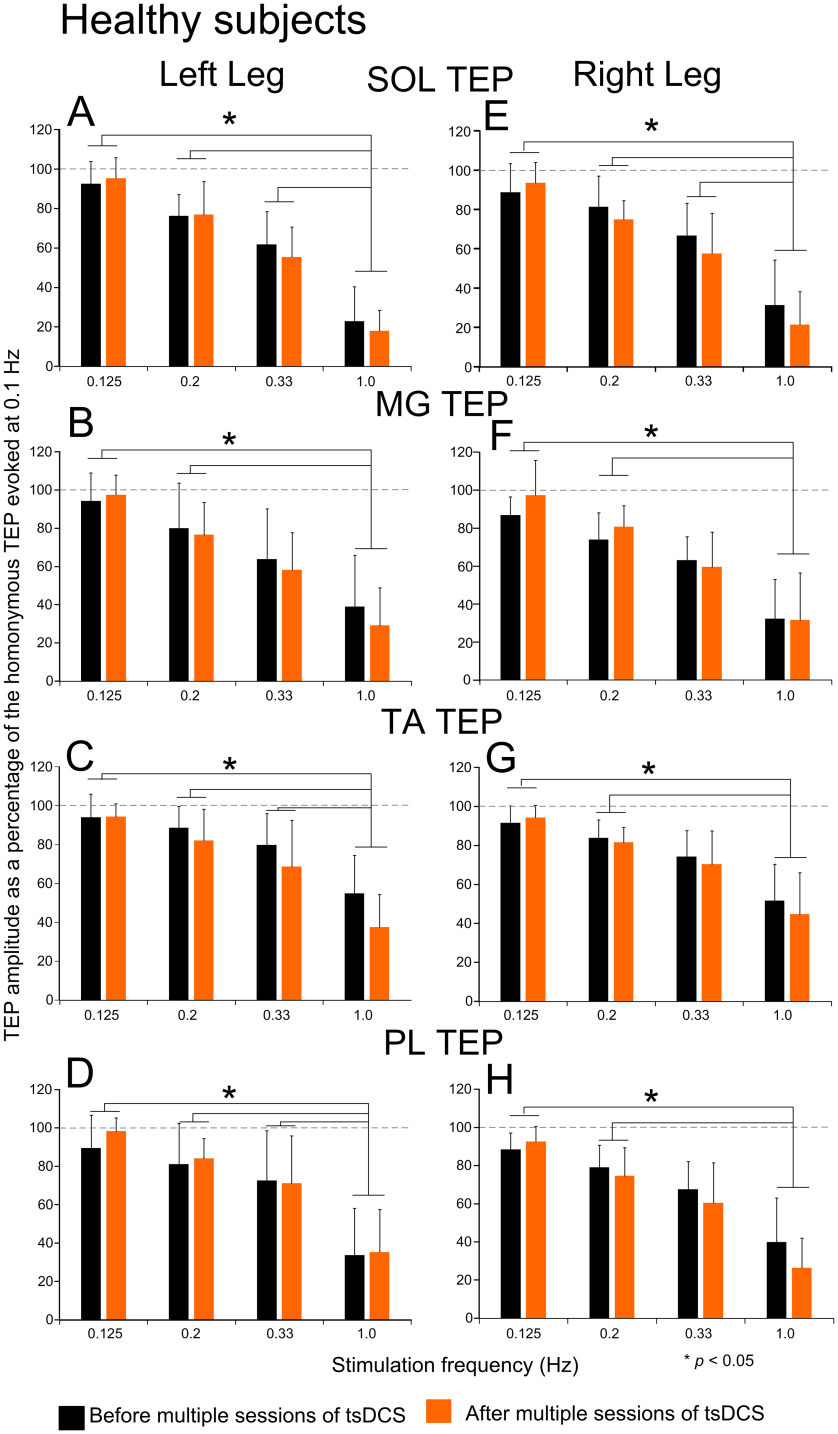
Homosynaptic depression before and after tsDCS in healthy subjects. Transspinal evoked potentials (TEPs) evoked at different stimulation frequencies before (black) and after (orange) multiple sessions of cathodal tsDCS are indicated the left and right SOL **(A, E)**, MG **(B, F)**, TA **(C, G)**, and PL **(D, H)** muscles for all healthy subjects. On the abscissa, the stimulation frequency is indicated. Ordinate indicates the TEPs amplitude normalized to the homonymous TEP evoked at a stimulation frequency of 0.1 Hz. Asterisks indicate statistically significant differences for TEPs evoked at different stimulation frequencies based on Bonferroni pairwise comparison results. No significant differences were found before and after tsDCS. Error bars denote the SD. SOL: soleus; MG: medial gastrocnemius; TA: tibialis anterior; PL: peroneus longus.

The TEPs homosynaptic depression from all *healthy subjects* and ankle muscles before and after tsDCS is indicated in Figure 7. Linear mixed model analysis showed that TEP amplitudes varied significantly as a function of stimulation frequency (*p* = 0.003), with all TEPs being significantly different largely at 1.0 Hz vs. 0.125 Hz and at 1.0 Hz vs. 0.2 Hz (*p* < 0.05; Bonferroni pairwise comparisons). These results support strong TEP frequency-dependent depression that was maximum at 1.0 Hz. However, the strength of homosynaptic depression did not change after tsDCS in the left SOL (F_2.81_ = 0.08, *p* = 0.79), left MG (F_2.79_ = 0.43, *p* = 0.55), left TA (F_2.77_ = 2.08, *p* = 0.25), left PL (F_2.79_ = 0.49, *p* = 0.53), right SOL (F_2.79_ = 0.37, *p* = 0.58), right MG (F_2.79_ = 0.11, *p* = 0.76), right TA (F_2.79_ = 0.11, *p* = 0.75), or right PL (F_2.79_ = 0.38, *p* = 0.58) TEPs. These results strongly support the inability of multiple sessions of tsDCS to alter the strength of TEPs homosynaptic depression in healthy subjects. Lastly, linear mixed model analysis showed no statistically significant differences among subject groups for the left SOL (F_4.72_ = 1.48, *p* = 0.316), left MG (F_4.72_ = 4.45, *p* = 0.08), left TA (F_4.72_ = 0.263, *p* = 0.77), left PL (F_4.72_ = 1.7, *p* = 0.27), right SOL (F_4.72_ = 1.94, *p* = 0.24), right MG (F_4.72_ = 1.32, *p* = 0.34), right TA (F_4.72_ = 1.96, *p* = 0.23), or right PL (F_4.72_ = 0.78, *p* = 0.51) TEPs recorded at baseline at different stimulation frequencies.

## 4 Discussion

This clinical trial bridged pathological expression and reorganization of spinal mechanisms following multiple sessions of tsDCS over the area of the spinal cord where the leg motor circuits reside in humans. We found that the net motor output changed for 10/16 muscles in AIS A-B, 8/16 muscles in AIS D, and 10/16 muscles in healthy subjects after multiple sessions of tsDCS. In human SCI, multiple sessions of tsDCS produced a complex modulation of the net spinal cord’s output with facilitation exerted mostly to distal ankle muscles and inhibition of proximal hip muscles. In healthy humans, multiple sessions of tsDCS produced a complex modulation pattern of the net motor output of spinal motor neurons bilaterally. tsDCS strengthened postactivation depression in AIS A-B but did not affect homosynaptic depression in any of the subject groups.

Motoneurons were recruited in sigmoidal order following single-pulse transspinal stimulation at increasing intensities over the thoracolumbar region in all subject groups, regardless of pathological sensation or movement (Figures 1–3), consistent with previous reports (36, 47). The sigmoid orderly recruitment applies to both corticospinal neurons and peripheral motor axons and signifies the non-linearity within a motoneuron pool and synaptic inputs to the pool (19, 48, 49). Transspinal stimulation transynaptically activates motoneurons in an orderly manner with increasing TEP amplitudes in all leg muscles from threshold to saturation (37, 38, 50). The TEPs recorded beyond 1.0 multiples of S50-TEPmax (Figures 1–3) cannot be interpreted simply as responses to synchronized Ia afferent excitatory synaptic events, as is the case for the H-reflexes, but rather as the difference between the net excitation and net inhibition acting on motoneurons and interneurons (19, 37). Therefore, TEPs at these intensities likely represent the net spinal motor output. This net spinal motor output was increased for ankle and knee muscles and decreased only for the left LH and VL muscles in AIS A-B neurological injuries (Figure 1). Similarly, in AIS D, the net spinal motor output increased for ankle and knee muscles and decreased for the VL and LH muscles (Figure 2). An increase in the net spinal motor output was also evident in participants with fully preserved sensation and movement, but the changes occurred in a more organized pattern with facilitation of the knee and most ankle flexors and inhibition of the knee extensors (Figure 3).

After SCI, spasticity manifests with increased motor output leading to spasms, co-contractions, clonus, and hyperreflexia but during movement, depolarization of motoneurons is unstable, which hinders movement performance. Thus, a beneficial therapeutic modality is the one that decreases hyperreflexia and spasticity while concomitantly increasing the net spinal motor output. Multiple sessions of transspinal stimulation with either direct or alternate current at low frequencies (0.2/30 Hz) depresses reflex hyperexcitability while concomitantly increasing motoneuron output of knee and ankle muscles in people with chronic SCI (19, 21, 51–52). Similar results have been reported in animal models, whereas single-pulse transspinal stimulation at alternating subthreshold-suprathreshold intensities profoundly decreased hyperreflexia and antagonist co-contractions and induced K+/Cl- cotransporter 2 membrane downregulation in lumbar motoneurons, preventing the development of spasticity in spinalized rats (53).

The changes we observed in the net output of spinal motoneurons 1–2 days after cessation of multiple sessions of tsDCS were likely driven by similar mechanisms of neuroplasticity in all subject groups. The electrical field produced by tsDCS extends a few spinal segments affecting both motor nuclei and spinal nerves, but fibers are more affected than the somata (41, 54). Cathodal direct current applied over the surface of the spinal cord depolarizes muscle spindle group Ia afferents and hyperpolarizes motor axons (55), but the same direct current can produce depolarization in some premotor nuclei and hyperpolarization in others (1). Further, computational modeling suggests that tsDCS affects the activity of spinal interneurons (56), which is consistent with the increases of aspartate, a glutamate analogue, and the effects reported on glycine and GABA receptor blockers in spinalized mice following cathodal tsDCS (9, 57). These neurotransmitters are engaged in the activity of several spinal interneuronal networks (58, 59). While findings from animals are not readily applicable to humans because of the effects of anesthetic drugs on spinal cord neuronal excitability (60), they do provide significant insights into potential mechanisms of action. Collectively, the effects we observed in the net motor output of spinal motoneurons might have resulted from changes in the depolarization strength of group Ia afferents, motor fibers, and synaptic inputs to spinal motoneurons and interneurons. Although recordings were taken on different days that may have resulted in different stimulation and recording sites, we marked the cathode transspinal stimulation electrode with a Tegaderm. However, given the complexity of the spinal neuronal networks involved in manifestation of TEPs, the specific mechanisms underlying the observed effects cannot be fully defined and more research is warranted to delineate the specific neuronal mechanisms at presynaptic and postsynaptic levels of motoneurons underlying multiple sessions of tsDCS.

Although TEPs homosynaptic depression at baseline was absent in AIS A-B but present in AIS D subjects (Figures 6, 7), no differences in the strength of TEPs homosynaptic depression was found among all three subject groups at the interstimulus intervals tested. This result raises two critical questions: is TEPs homosynaptic depression mediated by similar neuronal mechanisms to that of the soleus H-reflex and can TEPs homosynaptic depression be effectively used to probe neuroplasticity after an intervention? Homosynaptic depression, at least for the soleus H-reflex, is attributed largely to a decrease of the quanta of neurotransmitters at the Ia-motoneuron synapse released by the previously activated primary muscle spindle Ia afferents and manifested as decreased excitatory postsynaptic potentials (61–63). Homosynaptic depression is exerted at a presynaptic level but does not resemble the classical presynaptic inhibition that is accompanied by primary afferent depolarization and activity of spinal inhibitory interneurons (17). Most importantly, homosynaptic depression for the soleus H-reflex involves synchronized volleys of the same afferents and is stronger in small motoneurons (22). Taking into consideration the summation and occlusion of soleus TEPs with the soleus H-reflex (64) and the possibility that transspinal stimulation may evoke both antidromic and orthodromic afferent and motor volleys, homosynaptic depression of TEPs and the soleus H-reflex could not be ascribed to similar neuronal mechanisms.

Homosynaptic depression of the soleus H-reflex is pathological after SCI, leading to long periods of motoneuron depolarization and, thereby, hyperreflexia and clonus spasms after SCI (24, 25, 27). No systematic investigations in humans with and without SCI exist on spinal inhibitory mechanisms after multiple sessions of tsDCS. This clinical trial provided preliminary findings on daily sessions of tsDCS, which does not affect the strength of TEPs homosynaptic depression in any of the subject groups. These results are consistent with the inability of multiple sessions of tsDCS to affect the soleus H-reflex homosynaptic depression we recently reported for the same participants (52). Nonetheless, the TEPs and soleus H-reflex homosynaptic depression is profoundly potentiated following multiple sessions of single-pulse thoracolumbar transspinal stimulation in an alternating subthreshold-suprathreshold intensity arrangement in both SCI animal models and humans (19, 52, 53) as well as with transspinal stimulation at 30 Hz over the thoracolumbar region (21). This discrepancy is likely due to the underlying neuronal mechanisms of action with the two different stimulation modalities.

Postactivation depression for the right SOL and MG TEPs was substantially diminished or replaced by facilitation at baseline in AIS A-B neurological injuries when compared to that observed in healthy subjects, while it was of similar strength between AIS D and healthy subjects (compare Figures 4, 5). Daily sessions of tsDCS strengthened the right SOL and PL TEPs postactivation depression in AIS A-B at the interstimulus intervals of 60 and 100 ms (Figures 4E,H) and replaced depression with facilitation in healthy subjects at 300 and 500 ms for the right MG (Figure 5F). No significant effects were found in AIS D, which may be related to the pronounced depression present at baseline. Our findings that both homosynaptic and postactivation remained unchanged in AIS D after multi-session tsDCS support the use of alternative modalities to modulate spinal inhibitory mechanisms at iA-motoneuron synapse in these patients.

Postactivation depression of TEPs upon paired transspinal stimuli at 60 and 100 ms might also be mediated by the same mechanisms as homosynaptic depression (e.g., reduced transmitter release at the spinal level) because transcranial electric stimulation reduces the soleus H-reflex postactivation depression (56). The soleus H-reflex postactivation depression is maximal at 225 ms and recovery of the reflex amplitude starts at 300 ms (65), while facilitation has also been reported (65). The long latency effects point towards involvement of long-loop spinal reflex circuits (66, 67). Thus, the soleus H-reflex postactivation depression, brought up by paired stimuli, might be mediated by different neuronal mechanisms compared to homosynaptic depression. More research is needed on the nature of the inhibitory postsynaptic potentials associated with TEPs postactivation depression and homosynaptic depression. Evidence, however, supports the engagement of long corticospinal neuronal pathways. Ten sessions of tsDCS in healthy subjects decrease intracortical inhibition and increase intracortical facilitation and the slope of the right TA MEP recruitment input–output curve (68). Additionally, cathodal tsDCS has immediate effects on neuromodulation of the corticospinal motor drive (69) largely via the Ca^2+^ conductance that augments motoneuron activity (3).

All neurophysiological measures were taken 1–2 days after cessation of stimulation sessions. Therefore, long-term plasticity mechanisms, such as long-term potentiation and depression, morphological, anatomical, and histochemical changes, including changes in intrinsic firing properties and altered network properties, were likely involved (70, 71). This study, however, cannot pinpoint a specific neural mechanism mediating such plasticity, but daily sessions of tsDCS affected the net spinal motor output beyond 1.0 S50-TEPmax in people with SCI and the mechanisms that underly pathological expression of muscle tone and movement.

The main limitations of this clinical trial were the absence of a sham group and the imbalance of AIS grade neurological injuries. Participants with AIS grade C were not included due to an inability to recruit them, while one participant had AIS grade A injury, four participants had AIS grade B injury, and five participants had AIS grade D injury. Further, the time post-injury ranged significantly among participants (Table 1). Shorter time post-injury and higher levels of function at baseline are reported to respond better (72, 73) to activity-based therapeutic interventions.

In conclusion, tsDCS altered the net motor output of multiple spinal segments in all subject groups (AIS A-B, AIS D, and healthy subjects) and potentiated postactivation depression in AIS A-B but did not affect homosynaptic depression in any of the subject groups. This clinical trial is the first systematic investigation that supports the use of tsDCS as a potential neuromodulation strategy of spinal neuronal circuits and motoneuron excitability in people with SCI. The next step is to establish whether these changes are linked to functional improvements of movement.

## Data Availability

The original contributions presented in the study are included in the article/Supplementary material, further inquiries can be directed to the corresponding author.
