# Supplementary material for: Transspinal direct current stimulation as targeted therapy to increase motor neuron output and restore inhibition in human spinal cord injury
Source: Front Neurol. 2026 Mar 20;17:1773679. doi: 10.3389/fneur.2026.1773679 (PMC13047422; doi:10.3389/fneur.2026.1773679)
Supplement: Supplementary file 1 [file Table_1.docx]

**Supplementary Table 1**

**Inclusion/Exclusion Criteria**

*Inclusion criteria for people with spinal cord injury (SCI):*

- SCI above thoracic (T) 12 vertebra to ensure absent lower motoneuron lesion.
- Presence of tendon reflexes to be able to elicit the soleus H-reflex.
- A diagnosis of first time SCI due to trauma, vascular, or orthopedic pathology.
- Time post-SCI of more than 6 months.
- Age between 18 and 70 years.
- Stable medical condition without cardiopulmonary disease or cognitive impairment.
- Consent to participate in the study.
- Physician clearance is a pre-requisite for inclusion in the study.

*Inclusion criteria for people without SCI:*

- Intact sensation in both legs.
- Absent cardiopulmonary disease, musculoskeletal or neurological disorders.

*Exclusion criteria for people with SCI*:

- Supraspinal lesions.
- Neuropathies of the peripheral nervous system.
- Degenerative neurological disorders of the spine or spinal cord.
- Presence of pressure sores.
- Advanced urinary tract infection.
- Neoplastic or vascular disorders of the spine or spinal cord.
- Participation in a research study or out-patient rehabilitation program.

*Exclusion criteria for all participants:*

- Pregnant women or women who suspect they may be or may become pregnant will be excluded from participation because the risks of transspinal thoracolumbar stimulation to the fetus are unknown.
- People with cochlear implants, pacemakers and implanted stimulators of any type and purpose will be excluded to avoid their malfunction due to stimulation.
- Free of ferromagnetic material in the brain and/or spine.
- No contraindications to spinal stimulation.
